# Detection of Bovine Leukemia Virus RNA in Blood Samples of Naturally Infected Dairy Cattle

**DOI:** 10.3390/vetsci6030066

**Published:** 2019-08-06

**Authors:** Irene Alvarez, Natalia Gabriela Porta, Karina Trono

**Affiliations:** 1 Instituto de Virología, Instituto Nacional de Tecnología Agropecuaria (INTA), Buenos Aires C1686, Argentina; 2 Consejo Nacional de Investigaciones Científicas y Técnicas (CONICET), Godoy Cruz 2290, Ciudad autónoma de Buenos Aires C1425FQB, Argentina

**Keywords:** bovine leukemia virus, RNA detection, viral expression, tax gene, pol gene

## Abstract

The viral expression in vivo, in bovine leukemia virus (BLV)-infected cattle, is considered to be restricted to extremely low levels, and the mitosis of infected B lymphocytes is regarded as the main mode of virus persistence within the infected host. In this study, the presence of BLV RNA in whole blood from seven asymptomatic cows naturally infected with BLV during one year, including a complete milking cycle and two delivery time points, was investigated by nested-PCR using the oligonucleotides complementary to the tax and pol gene. BLV RNA was detected in four cows at different time points, especially in high blood proviral load cows and around delivery time. This study describes for the first time the detection of free BLV RNA in blood from BLV-infected asymptomatic cows. The results obtained suggest the occurrence of persistent low-level expression of the tax and pol genes that could be a result of viral reactivation, within the asymptomatic period. This finding may be important in the pathogenesis of BLV infection, associated with the delivery period.

## 1. Introduction

In Argentina, milking cows from high production areas are highly infected with the bovine leukemia virus (BLV) with values of individual prevalence as high as 90% [1]. This is in line with findings from the United States and Canada [2,3]. 

Although the majority of the BLV-infected animals course the infection asymptomatically, dairy farms are negatively impacted by deaths from lymphosarcoma occurring in 5–10% of lactating cows, resulting in high-profit losses [4]. 

Since the expression of BLV in vivo is considered to be extremely low, the mitosis of infected B lymphocytes is regarded as the main mode of virus persistence within the infected host [5]. BLV RNA or antigens have been detected in leukocytes isolated from blood in tumor cells, but have not been detected in fresh blood, even when the transcription is activated ex vivo rapidly after blood extraction. Hence, the blood proviral load (PVL) is currently considered as a marker of infection, useful for monitoring and prediction of the potential transmission in cattle within herds. Therefore, animal segregation as a control strategy according to in vivo levels of PVL depends both on the stability of this marker and on the absence of circulating virus.

Few studies have addressed the importance of BLV RNA detection in naturally infected cows and the relationship between viral load and the clinical implications [6,7]. In this study, we investigated, at different time points, the presence of BLV RNA in whole blood from seven asymptomatic dairy cows naturally infected with BLV during one year, including a complete milking cycle and two delivery events.

## 2. Materials and Methods 

### 2.1. Samples under Study

Seven cows from a commercial dairy farm naturally infected with BLV (80% of individual prevalence), in the second or third milking cycle, were chosen. Whole blood samples were obtained during a one-year period between two delivery events, including a complete milking cycle at different time points: five days before delivery, delivery (D), five days after delivery (5 AD), two months (2 M), four months (4 M), six months (6 M), eight months (8 M), ten months (10 M) after delivery, five days before second delivery (5 BD2), second delivery (D2). The procedures followed for extraction and handling of samples were reviewed and approved by the Institutional Committee for Care and Use of Experimental Animals of the National Institute of Agricultural Technology (CICUAE-INTA, Buenos Aires, Argentina), following the guidelines described in The Institutional Manual. All blood samples were mixed with the RNA stabilizer solution (RNAlater, AMBION, ThermoFisher, Waltham, MA, USA) immediately after collection to stabilize RNA and to prevent the ex-vivo BLV viral expression. Plasma samples were separated by centrifugation of a whole blood fraction and stored at −20 °C. 

### 2.2. Nucleic Acid Purification 

Total RNA was extracted from whole blood using the commercial kit High Pure RNA Purification Kit (Roche, Penzberg, Germany), following the manufacturer’s instructions. RNA samples were treated with RQ1 DNAse enzyme (Promega, Madison, WI, USA) to eliminate DNA contamination. Total genomic DNA was extracted from buffy coat using the High Pure PCR Template Preparation Kit (Roche, Penzberg, Germany), according to the manufacturer’s instructions.

### 2.3. Nucleic Acid Amplification

BLV RNA was detected after cDNA synthesis using random hexamers by nested-PCR using the oligonucleotides, previously described [8], complementary to the *tax* gene in the BLV genome: 7781F (5′-CAGACACCAGGGGAGCCATA-3′) and 8083R (5′-CTGCTAGCAACCAATTTCGGA-3′) for the first round and 7802F (5′-AGCCATACGTTATCTCTCCA-3′) and 8062R (5′-CAGGTTAGCGTAGGGTCATG-3′) for the second round. We also used oligonucleotides complementary to the *pol* gene in the BLV genome [9] pol2846F (5′-ATGTTATCAAGCCCTGGCTGC-3′) and pol3319R (5′-CTTGGTTGTCAGTCAAGG-3′) for the first round, and pol2984F (5′-CTACCTTGCAGATCTCATC-3′) and pol3164R (5′-GCTTGTCGAAGCTCTGCAATGC-3′) for the second round. Each treated RNA sample was used as a direct template sample in both nested-PCR assays to detect potential DNA contamination. DNA positive and negative controls were included in each run test. The BLV tax gene amplification produced a single band of 280 bp, and the pol gene produced a single band of 200 bp. All samples were analyzed in duplicate, in two independent runs. 

To determine if the animals were positive for proviral BLV, DNA samples from buffy coat were analyzed by nested-PCR using the same oligonucleotides mentioned above.

PVL was quantified by a real-time quantitative PCR (qPCR), as previously reported [10,11,12]. Briefly, the SybrGreen chemistry was used, amplifying a region of 120 bp of the pol gene and 500 ng of DNA template. The specificity of each reaction was confirmed by dissociation curve analysis. As standard, a plasmid pBLV1 (provided by Jacek Kuzmak, National Veterinary Research Institute, Pulawy, Poland) containing BLV pol fragment was used. Ten-fold dilutions of this standard were made from 1 × 10^6^ to 1 copy/μL. A strong and weak positive control and 2 negative controls were included in each plate. The limit of detection of the assay was 5 BLV copies per reaction (10 BLV copies/μg of DNA). According to our own previously defined criteria, the proviral load was declared as high when it exceeded 1500 copies/µg of DNA.

### 2.4. BLV Serology

To detect serological response to BLV, plasma samples of all animals at all time points were analyzed by ELISA, as previously reported [13]. 

## 3. Results

**Figure 1:**
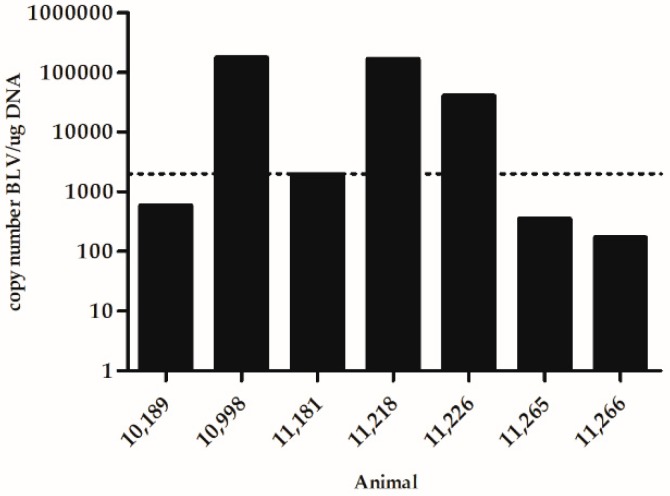
Blood proviral load (PVL) in the peripartum period in cows under study. Dot line corresponds to the threshold between high and low PVL.

Cows were classified according to their circulating blood PVL in the peripartum period using a quantitative PCR (qPCR) (Figure 1). Cows number 10,998, 11,226, and 11,218 were considered as High PVL (>1,5000 copies/µg of DNA), and cows 10,189, 11,181, 11,265, and 11,266 were considered as Low PVL.

**Figure 2:**
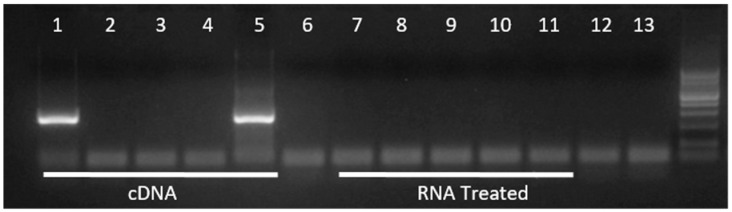
Detection of BLV (bovine leukemia virus) tax gene by nested-PCR. Amplification of cDNA obtained by retrotranscription (RT) reaction from isolated and treated RNA; each treated RNA sample was used also as a direct template to control the DNA contamination. (1), (7): 11,218—Date of delivery; (2), (8): 10,998—Date of delivery; (3), (9): 11,226—Date of delivery; (4), (10): 10,189- Date of delivery; (5), (11): 11,181- Date of delivery; (6) Non- template control of RT; (12) Non- template control of first-round PCR; (13) Non- template control of second-round PCR.

BLV RNA was detected in whole blood samples of four cows at different time points (Table 1) (Figure 2). BLV proviral DNA contamination was not detected in any sample analyzed when using extracted and treated RNA as a template (Figure 2). In cows with high blood PVL in the peripartum period (10,998, 11,226, and 11,218), BLV RNA was detected around delivery time and also during the milking cycle at 4 and 8 months after delivery. In cow 11,181, BLV RNA was detected only around delivery. In this cow, only the tax gene amplification was detectable, as well as in cow 10,998. 

All animals were seropositive along the whole milking cycle, and also all cows tested positive for proviral BLV DNA in whole blood at all sampling times using nested-PCR assay for both *tax* and *pol* genes. A supplementary data file was added with all Nested-PCR and serology results from animals used in this study (Table S1).

## 4. Discussion

This study describes the detection of BLV RNA in whole blood from the asymptomatic dairy cows naturally infected with BLV, suggesting the occurrence of some kind of BLV reactivation and/or replication within the asymptomatic period.

During BLV infection, genomic RNA acts as a template for the production of transcripts for structural and enzymatic proteins (gag, pol, and env) and also transcripts for regulatory proteins (Tax, Rex, R2, G4) [14,15]. It was previously considered that established BLV infection occurs in the absence or at very low levels of viral expression since BLV virions, transcripts, or viral structural proteins cannot be directly detected in peripheral blood or tumor by conventional standard techniques [5]. However, the persistent presence of antibodies to viral proteins suggest a continuous, low-level production of viral proteins. On the other hand, BLV tax/rex transcripts have been detected in blood and lymphosarcoma tissues from BLV-infected cattle and sheep [7,8,14,16]. Using the RT-PCR technique, different transcription patterns are discovered in the different pathogenic stages of infection with BLV in animals with long-time infection or with tumors [15,17,18].

In BLV-infected cattle, the expression of BLV can be activated in anticoagulated whole blood or purified leukocytes upon incubation at 37 °C without exogenous factors [19,20]. To reduce this ex vivo expression, the whole blood samples used in this study were taken in the presence of RNA stabilizer and protector, and the detection of BLV transcripts could be indicative of a viral reactivation/expression event associated with some factor present during the delivery time.

RNA of tax BLV gene was detected in four animals regardless of the milking cycle time, suggesting that persistent low-level expression of the tax gene may be important in the pathogenesis of BLV infection, due to the transactivating function of Tax on BLV infection. The detection of the pol gene RNA in only two animals could be a result of viral reactivation associated with the delivery period. The estimated detection limit of both nested-PCR assays used is about one infected cell in 10,000 non-infected cells.

BLV induces a life-long persistent infection in cattle, with permanent antibody response, where the majority of infected cattle remains asymptomatic. Since in most of the infected animals, BLV DNA is detected in peripheral blood as a provirus, blood PVL is the only virological marker available for disease monitoring during BLV infection. The presence of BLV-infected animals with high PVL are dangerous for a dairy herd because a high level of BLV infection is associated with a greater transmission probability [21,22]. In the present study, animals with high PVL at the start point of the study (10,998–11,218–11,226) might have a higher potential risk of BLV transmission due to the intermittent evidence of BLV transcripts in blood samples, particularly around delivery. This finding was also observed in human T-lymphotropic virus (HTLV) infection where patients with high PVL have a greater risk of developing myelopathy (HAM/TSP) or adult T-cell leukemia/lymphoma (ATLL) and of transmitting HTLV horizontally by milk [23]. 

Viral load is commonly used as a marker of disease progression in retroviral infections because it indicates the degree of viral replication. In patients infected with HIV-1, the plasma viral load is directly related with the progression of the disease and is an important surrogate marker in predicting the development of acquired immunodeficiency syndrome (AIDS) [24]. In HTLV-1 infection, the detection of HTLV-1 RNA by RT-PCR in plasma samples of asymptomatic carriers and patients with HAM/TSP has been reported, but always at a very low copy number [25,26].

Since the detection of BLV RNA was performed on whole blood samples, it was not possible to discriminate if these results correspond to free RNA in plasma or to cell-associated BLV RNA. Depending on whether it is one or the other, the potential risk and its implications in BLV infection would be different and should be studied. Also, it must be determined if the detection of BLV RNA corresponds only with the detection of transcripts or the detection of infectious virus. Considering these possibilities, other transmission pathways, rather than cell-related contact, should be further investigated in natural BLV infection.

In summary, this study highlights the detection of BLV RNA in whole blood from the asymptomatic cows naturally infected with BLV at different time points during a complete milking cycle. Therefore, our results suggest that BLV viral expression, reactivation, and/or replication could be present during the asymptomatic period in the naturally BLV-infected animals. This RNA detection appears to be associated with the delivery time, especially in high blood PVL cows. Even if we consider that in this study, BLV RNA was only rarely detected in blood samples, more animals with different blood PVL and in their first lactation cycle should be further investigated to confirm these findings. More studies are also warranted to determine whether the detection of BLV RNA in whole blood could be used as a marker of disease progression or infection risk. To this end, a specific and sensitive BLV RNA quantitative PCR assay is currently being standardized in our group.

## Figures and Tables

**Table 1:** BLV (bovine leukemia virus) RNA detection by RT-nested PCR in whole blood samples of naturally infected animals. Tax and pol genes were amplified. (+: positive; −: Not detected; NE: Not evaluated). All samples were analyzed in duplicate.

	High PVL ^2^						Low PVL ^2^							
Time Point ^1^	10,998		11,218		11,226		10,189		11,181		11,265		11,266	
	Tax	Pol	Tax	Pol	Tax	Pol	Tax	Pol	Tax	Pol	Tax	Pol	Tax	Pol
5 BD	+	−	−	−	−	−	−	−	−	−	−	−	−	−
D1	−	−	+	+	−	−	−	−	+	−	NE	NE	−	−
5 AD	NE	NE	+	−	−	−	−	−	−	−	−	−	−	−
2 M	−	−	−	−	−	−	−	−	−	−	−	−	−	−
4 M	+	−	−	+	−	−	−	−	−	−	−	−	−	−
6 M	−	−	−	−	−	−	−	−	−	−	−	−	−	−
8 M	−	−	+	+	−	−	NE	NE	−	−	−	−	−	−
10 M	−	−	−	−	−	−	NE	NE	−	−	−	−	−	−
5 BD2	−	−	−	−	NE	NE	NE	NE	−	−	−	−	NE	NE
D2	+	−	+	−	+	+	NE	NE	−	−	−	−	NE	NE

^1^ 5 BD: 5 days before delivery, D: date of delivery, 5 AD: 5 days after delivery, M: months after delivery, 5 BD2: five days before second delivery, D2: second delivery. ^2^ Proviral load (PVL) determined at the start point of the study, 2 months before the delivery 1.

## Supplementary Materials

The following are available online at https://www.mdpi.com/2306-7381/6/3/66/s1, Table S1: Nested-PCR and serology results from animals used in this study. (pos: positive; neg: Not detected; NE: Not evaluated). All samples were analyzed in duplicate.
