# Correction: Alvarez, I., et al. Detection of Bovine Leukemia Virus RNA in Blood Samples of Naturally Infected Dairy Cattle. *Vet. Sci.* 2019, *6*, 66

**DOI:** 10.3390/vetsci6040084

**Published:** 2019-10-23

**Authors:** Irene Alvarez, Natalia Gabriela Porta, Karina Trono

**Affiliations:** 1 Instituto de Virología, Instituto Nacional de Tecnología Agropecuaria (INTA), Buenos Aires C1686, Argentina; 2 Consejo Nacional de Investigaciones Científicas y Técnicas (CONICET), Godoy Cruz 2290, Ciudad autónoma de Buenos Aires C1425FQB, Argentina

The authors wish to make the following corrections to this paper [1]:

In the abstract, the sentence “This study describes for the first time the detection of free BLV RNA in blood from BLV-infected asymptomatic cows” should be “This study describes the detection of free BLV RNA in blood from BLV-infected asymptomatic cows”.

In the discussion section, we eliminated the sentence “Therefore, our results suggest that BLV viral expression, reactivation, and/or replication could be present during the asymptomatic period in the naturally BLV-infected animals”, and added the sentence “This is in line with recently reported data [Jaworski et al., BMC Vet Res 2019, 15, 150], and reinforces the evidence suggesting that BLV viral expression, reactivation and/or replication could be present during the asymptomatic period in naturally BLV-infected animals”.

Also, a new reference was added [2]: Jaworski, J.P.; Petersen, M.I.; Carignano, H.A.; Trono, K.G. Spontaneous virus reactivation in cattle chronically infected with bovine leukemia virus. BMC Vet Res 2019, 15, 150.

The authors would like to apologize for any inconvenience caused to the readers by these changes.
