# Supplementary material for: Detection of Bovine Leukemia Virus RNA in Blood Samples of Naturally Infected Dairy Cattle
Source: Vet Sci. 2019 Aug 6;6(3):66. doi: 10.3390/vetsci6030066 (PMC6789540; doi:10.3390/vetsci6030066)
Supplement: Supplementary file 1 [file vetsci-06-00066-s001.pdf]

Supplementary data

Table S1. Nested-PCR and serology results from animals used in this study. (pos: positive; neg: Not detected; NE: Not evaluated). All samples were analyzed in duplicate.

ID Animal	Time point (1)	nested-PCR tax BLV			nested-PCR pol BLV			Serology anti BLV (ELISA)
		cDNA	RNA-treated	provial DNA	cDNA	RNA-treated	provial DNA	
**10998**	5AP	pos	neg	pos	neg	neg	pos	pos
	P	neg	neg	pos	neg	neg	pos	pos
	5PP	NE	NE	pos	NE	NE	pos	pos
	2M	neg	neg	pos	neg	neg	pos	pos
	4M	pos	neg	pos	neg	neg	pos	pos
	6M	neg	neg	pos	neg	neg	pos	pos
	8M	neg	neg	pos	neg	neg	pos	pos
	10M	neg	neg	pos	neg	neg	pos	pos
	5AP2	neg	neg	pos	neg	neg	pos	pos
	P2	pos	neg	pos	neg	neg	pos	pos
**11218**	5AP	neg	neg	pos	neg	neg	pos	pos
	P	pos	neg	pos	pos	neg	pos	pos
	5PP	pos	neg	pos	neg	neg	pos	pos
	2M	neg	neg	pos	neg	neg	pos	pos
	4M	neg	neg	pos	pos	neg	pos	pos
	6M	neg	neg	pos	neg	neg	pos	pos
	8M	pos	neg	pos	pos	neg	pos	pos
	10M	neg	neg	pos	neg	neg	pos	pos
	5AP2	neg	neg	pos	neg	neg	pos	pos
	P2	pos	neg	pos	neg	neg	pos	pos
**11226**	5AP	neg	neg	pos	neg	neg	pos	pos
	P	neg	neg	pos	neg	neg	pos	pos
	5PP	neg	neg	pos	neg	neg	pos	pos
	2M	neg	neg	pos	neg	neg	pos	pos
	4M	neg	neg	pos	neg	neg	pos	pos
	6M	neg	neg	pos	neg	neg	pos	pos
	8M	neg	neg	pos	neg	neg	pos	pos
	10M	neg	neg	pos	neg	neg	pos	pos
	5AP2	NE	NE	pos	NE	NE	pos	pos
	P2	pos	neg	pos	pos	neg	pos	pos
**10189**	5AP	neg	neg	pos	neg	neg	pos	pos
	P	neg	neg	pos	neg	neg	pos	pos
	5PP	neg	neg	pos	neg	neg	pos	pos
	2M	neg	neg	pos	neg	neg	pos	pos
	4M	neg	neg	pos	neg	neg	pos	pos
	6M	neg	neg	pos	neg	neg	pos	pos
	8M	NE	NE	pos	NE	NE	pos	pos
	10M	NE	NE	pos	NE	NE	pos	pos
	5AP2	NE	NE	pos	NE	NE	pos	pos

	P2	NE	NE	pos	NE	NE	pos	pos
**11181**	5AP	neg	neg	pos	neg	neg	pos	pos
	P	pos	neg	pos	neg	neg	pos	pos
	5PP	neg	neg	pos	neg	neg	pos	pos
	2M	neg	neg	pos	neg	neg	pos	pos
	4M	neg	neg	pos	neg	neg	pos	pos
	6M	neg	neg	pos	neg	neg	pos	pos
	8M	neg	neg	pos	neg	neg	pos	pos
	10M	neg	neg	pos	neg	neg	pos	pos
	5AP2	neg	neg	pos	neg	neg	pos	pos
	P2	neg	neg	pos	neg	neg	pos	pos
**11265**	5AP	neg	neg	pos	neg	neg	pos	pos
	P	NE	NE	pos	NE	NE	pos	pos
	5PP	neg	neg	pos	neg	neg	pos	pos
	2M	neg	neg	pos	neg	neg	pos	pos
	4M	neg	neg	pos	neg	neg	pos	pos
	6M	neg	neg	pos	neg	neg	pos	pos
	8M	neg	neg	pos	neg	neg	pos	pos
	10M	neg	neg	pos	neg	neg	pos	pos
	5AP2	neg	neg	pos	neg	neg	pos	pos
	P2	neg	neg	pos	neg	neg	pos	pos
**11266**	5AP	neg	neg	pos	neg	neg	pos	pos
	P	neg	neg	pos	neg	neg	pos	pos
	5PP	neg	neg	pos	neg	neg	pos	pos
	2M	neg	neg	pos	neg	neg	pos	pos
	4M	neg	neg	pos	neg	neg	pos	pos
	6M	neg	neg	pos	neg	neg	pos	pos
	8M	neg	neg	pos	neg	neg	pos	pos
	10M	neg	neg	pos	neg	neg	pos	pos
	5AP2	NE	NE	pos	NE	NE	pos	pos
	P2	NE	NE	pos	NE	NE	pos	pos

[1]. 5BD: 5 days before delivery, D: date of delivery, 5AD: 5 days after Delivery, M: months after delivery, D2: second delivery.
